# Strengthening Patient-Safety in ERAS Pathways: An Evidence-Informed Framework for Simulation-Based Nursing Practice Development in Acute Surgical Care

**DOI:** 10.3390/healthcare14101317

**Published:** 2026-05-12

**Authors:** Ramasubbamma Ramaiah, Eva Lobelle Sampayan, Rasha Elsayed Ahmed, Enas A. Assaf, Ester Mary Pappiya, Kalaiselvi Duraisamy, Mathar Mohideen Nagoor Thangam, Logapriya Rajagopal Sambasivan, Deepak Jayapal, Pavithra Jayapal, Krishnaraju Venkatesan, Mervat Mostafa Arrab

**Affiliations:** 1 College of Nursing, Khamis Mushait, King Khalid University, Abha 61413, Saudi Arabia; esampayan@kku.edu.sa (E.L.S.); raslsalem@kku.edu.sa (R.E.A.); eassaf@kku.edu.sa (E.A.A.); kalaiselvi@kku.edu.sa (K.D.); marb@kku.edu.sa (M.M.A.); 2 College of Nursing, Tanta University, Tanta 31527, Egypt; 3 Department of Maternity and Child Health Nursing, College of Nursing, Najran University, Najran 61441, Saudi Arabia; empappiya@nu.edu.sa; 4 Department of Medical Surgical Nursing, Faculty of Nursing, University of Tabuk, Tabuk 71491, Saudi Arabia; mthangam@ut.edu.sa; 5 Government Medical College, Thiruvallur, TN Dr. MGR Medical University, Chennai 602024, India; rslogapriya@gmail.com (L.R.S.); deepakjayapal1989@gmail.com (D.J.); 6 Command Hospital Airforce, Rajive Gandhi University of Health Science, Bengaluru 560041, India; pavithra.13june@gmail.com; 7 Department of Pharmacology, College of Pharmacy, King Khalid University, Abha 61413, Saudi Arabia; kvenkatesan@kku.edu.sa

**Keywords:** enhanced recovery after surgery, simulation training, education, nursing, baccalaureate, patient-safety, medical–surgical nursing

## Abstract

**Background:** Enhanced Recovery After Surgery (ERAS) pathways depend on nursing-led safety behaviours such as early mobilisation, opioid-sparing analgesia, device minimisation, and reliable discharge teaching to prevent immobility-related, opioid-related, and device-related harms. However, pre-licensure medical–surgical preparation inconsistently embeds these competencies, leaving ERAS delivery and patient-safety vulnerable to variation. **Objective:** To develop an evidence-informed, practice-development framework that translates ERAS principles into measurable nursing competencies and management priorities explicitly linked to patient-safety, quality improvement, and harm reduction in acute surgical care. **Methods:** This practice-development framework paper used a narrative literature review of ERAS guidelines, AACN Essentials, and published simulation reports (MEDLINE, CINAHL, Embase, Scopus, ERIC) to identify recurring competencies and scenario features. These were inductively organised and mapped to patient-safety priorities to derive a four-domain framework. **Findings:** Identified simulations emphasised early mobilisation and multimodal analgesia; nutrition, fluid stewardship, device minimisation, and ERAS-focused patient education were less represented. High-fidelity and virtual formats improved knowledge and confidence but rarely reported patient-level outcomes. These gaps informed a four-domain framework: (1) ERAS clinical pillars and priority nursing competencies; (2) scenario and modality design (including a worked POD-1 colorectal case); (3) assessment and feedback strategies anchored by the Lasater Clinical Judgement Rubric; and (4) implementation tools for nurse managers and ERAS leads to integrate simulation into orientation and quality dashboards. The framework conceptually links competencies to safety-relevant endpoints including opioid-related adverse events, immobility-related complications, device-related harms, and discharge-education reliability. **Conclusions:** ERAS-aligned simulation may offer a feasible, scalable patient-safety and practice-development strategy for aligning pre-licensure preparation with nursing-management priorities for harm reduction. The framework provides a conceptual model that warrants empirical evaluation. It maps ERAS pillars to nursing competencies, operationalises these through a reusable colorectal scenario, and links simulation-derived competencies to unit-level recovery and safety agendas.

## 1. Introduction

Major surgery carries risks of pain, ileus, cardiopulmonary instability, thromboembolic events, and delayed functional recovery, many of which represent preventable or reducible harms when evidence-based perioperative pathways are applied consistently. Enhanced Recovery After Surgery (ERAS) standardises perioperative care to reduce these complications through early oral intake, multimodal and opioid-sparing analgesia, early mobilisation, and goal-directed fluid therapy [1]. Although ERAS has been shown to improve clinical outcomes and reduce length of stay, its successful implementation depends heavily on reliable nursing behaviours that directly influence patient-safety, including early mobilisation, opioid stewardship, fluid and nutrition optimisation, device minimisation, and discharge teaching [2,3].

However, nursing practice-development structures, including pre-licensure medical–surgical preparation and unit-based competency systems, do not consistently reflect contemporary ERAS principles [4]. Consequently, many nurses lack structured opportunities to develop the clinical judgement, interprofessional communication, and patient coaching competencies needed to protect patients from immobility-related complications, opioid-related adverse events, device-related harms, and unsafe discharge [5]. From a patient-safety perspective, this preparation gap contributes to avoidable variation in ERAS adherence and preventable harm profiles across organisations [6].

Simulation-based education offers a viable strategy to bridge this gap [7,8]. In controlled environments, pre-licensure and early-career nurses can rehearse ERAS-aligned safety behaviours, such as mobilising a postoperative patient, titrating multimodal analgesia, clarifying erroneous NPO orders, and teaching discharge instructions using teach-back, without exposing patients to risk. Recent empirical evidence supports the effectiveness of simulation for developing clinical reasoning, teamwork, and protocol adherence in nursing students [9,10]. When designed as part of an ERAS implementation strategy, simulation can also be used with existing staff to standardise communication patterns, test local processes, and generate observable data for quality improvement.

The conceptual pathway underpinning this framework follows three steps. First, ERAS guidelines specify nursing-sensitive safety behaviours, including early mobilisation, opioid-sparing analgesia, fluid stewardship, device minimisation, and reliable discharge teaching. Second, these behaviours require clinical judgement, interprofessional communication, and patient coaching competencies that are observable and assessable. Third, simulation provides a controlled environment for rehearsing these behaviours, receiving structured feedback, and demonstrating competency before clinical exposure. This pathway translates ERAS principles from guidelines into measurable nursing actions and simulation design elements, directly linking educational activities to patient-safety and quality-improvement goals.

In response, this paper proposes an evidence-informed, practice-development framework that embeds ERAS principles within simulation-enabled learning for pre-licensure nurses and early-career staff. The framework is organised into four domains: (1) ERAS clinical pillars and priority nursing competencies; (2) scenario and modality design; (3) assessment and feedback strategies; and (4) implementation tools for nurse managers and ERAS programme leads. Unlike prior publications that focus on staff training or general perioperative competencies [11,12], this framework explicitly links simulation-derived competencies to patient-safety endpoints including opioid-related adverse events, immobility-related complications, device-related harms, and discharge-education reliability, and provides reusable, AACN Essentials-aligned tools for clinical practice development.

The significance of this practice-development shift can be illustrated briefly. Under traditional postoperative care, a patient may remain NPO for days, receive high-dose opioids, and stay bedbound—practices that increase risks of ileus, venous thromboembolism, and prolonged hospitalisation [13,14,15]. In contrast, an ERAS approach promotes early oral fluids, multimodal analgesia, and mobilisation on postoperative day one, which are associated with earlier recovery and fewer complications [14]. For nurses, ERAS requires a shift from task-oriented routines to proactive, physiology-informed, recovery-oriented actions. Embedding these competencies in simulations offers a structured, equitable method to prepare nurses for ERAS-driven care and to make safety-critical behaviours observable for feedback and quality monitoring.

This framework was developed using an evidence-informed, practice-development design process (detailed in Section 2). Briefly, we conducted a narrative literature review of ERAS guidelines, AACN Essentials, and published simulation reports involving pre-licensure nursing students. Recurring competencies and scenario features were inductively organised, mapped to patient-safety priorities, and refined through iterative input from nurse managers, ERAS clinical leads, and educators. The resulting framework is positioned as a conceptual model that warrants empirical evaluation; it does not report findings from a systematic review or provide definitive evidence of patient-safety outcomes.

In summary, this practice paper aims to translate core ERAS elements into nursing practice competencies explicitly linked to patient-safety and recovery reliability; to propose simulation scenarios, assessment tools, and workflow supports that operationalise these competencies for pre-licensure learners and early-career staff; and to outline feasible implementation and evaluation strategies that nurse managers and ERAS programme leads can adapt for local quality-improvement agendas. By foregrounding safety-relevant endpoints and providing concrete, transferable tools, the framework seeks to strengthen the reliability of acute postoperative care and intends to reduce preventable harm across ERAS pathways.

## 2. Methods

This paper was developed as an evidence-informed narrative review and framework-development article rather than a systematic review. Literature identification was purposive and iterative, drawing on ERAS Society guidance, AACN Essentials documents, and targeted searches of MEDLINE, CINAHL, Embase, Scopus, and ERIC for simulation and experiential-learning reports relevant to pre-licensure nursing and postoperative or ERAS-related care. Because the aim was conceptual framework development rather than exhaustive evidence aggregation, sources were used to identify recurring themes, competency targets, and illustrative exemplars. No formal study inventory, PRISMA flow diagram, or quantitative synthesis was undertaken, and the references cited in this paper should not be interpreted as a systematically derived set of included studies.

The framework development process comprised three overlapping steps: narrative literature review, iterative expert input, and inductive synthesis. The targeted searches covered January 2000 to December 2025 and used terms related to enhanced recovery (e.g., “enhanced recovery after surgery,” “perioperative pathway”), simulation (e.g., “simulation,” “high-fidelity,” “virtual scenario”), and pre-licensure nursing education. Sources considered relevant included ERAS Society guidelines, AACN Essentials documents, and published reports describing simulation or experiential-learning activities involving pre-licensure nursing students with postoperative or ERAS-related content. Opinion pieces without direct relevance to framework development and non-English articles were excluded. No formal risk-of-bias appraisal was performed.

The framework was then refined through iterative input from content experts. The author team (n = 12) includes specialists in ERAS implementation, medical–surgical nursing, simulation pedagogy, nursing management, and patient-safety. In addition, three external nurse managers and two clinical educators from ERAS-implementing hospitals reviewed the draft framework. Feedback was incorporated through iterative discussion and thematic consideration of comments. This expert review process was intended to strengthen clinical relevance, pedagogical plausibility, and implementation feasibility rather than to generate formal consensus recommendations. Because no structured consensus method, such as Delphi, nominal group technique, or rating-based agreement process, was used, the final framework should be interpreted as expert-informed rather than consensus-validated.

Finally, recurring competencies, scenario features, and reported educational out-comes identified through the narrative review were inductively organised and mapped to ERAS guidelines and the AACN Essentials. This process yielded four interconnected domains: (1) ERAS clinical pillars and priority nursing competencies; (2) scenario and modality design; (3) assessment and feedback strategies; and (4) implementation tools for nursing management and quality improvement. The representative postoperative day-one colorectal scenario and Lasater Clinical Judgement Rubric-based assessment tools were developed as conceptual examples rather than validated instruments.

This approach has important limitations, which are further elaborated in the Discussion (Section 4.4). The literature identification process was purposive rather than systematic, and no formal source enumeration or reproducible included-study inventory was generated. Expert input strengthened the framework’s clinical and educational relevance, but the absence of a structured consensus process limits claims of formal agreement. Accordingly, the framework should be interpreted as evidence-informed and practice-oriented, not as an empirically validated or consensus-validated model.

## 3. Results

The narrative review and expert-informed refinement process generated two principal outputs. First, the reviewed literature and guideline documents converged on recurrent ERAS-related nursing safety behaviours that were sufficiently consistent to serve as anchors for framework development. Second, these behaviours were translated into a four-domain practice-development framework encompassing competency mapping, scenario design, assessment strategies, and implementation supports. The results presented below should therefore be interpreted as outputs of a conceptual framework-development process rather than as empirically validated intervention effects.

### 3.1. Literature-Informed Identification of Core ERAS Nursing Safety Behaviours

ERAS programmes emphasise a coordinated bundle of evidence-based, patient-safety-relevant interventions that depend heavily on nursing assessment, patient education, and interprofessional communication [15]. Nurses act as advocates, monitors, and care coordinators throughout the perioperative continuum [16]. To support reliable ERAS implementation and reduce preventable harm, the following six safety behaviours are defined as core nursing responsibilities:

- Early mobilisation—initiating and assisting progressive ambulation within ERAS timeframes. Safety rationale: prevents immobility-related complications including venous thromboembolism, atelectasis, pneumonia, pressure injuries, and muscle deconditioning [17,18,19].
- Opioid-sparing multimodal analgesia—performing structured pain assessments; implementing non-opioid and non-pharmacologic strategies first; using opioids judiciously. Safety rationale: reduces opioid-related adverse events (ileus, oversedation, delirium, respiratory depression) while maintaining functional pain control [20,21,22].
- Early oral feeding—assessing readiness to advance diet; initiating ordered early feeding; monitoring tolerance. Safety rationale: supports return of gut function; reduces infection and ileus; prevents prolonged NPO-related complications [7,23,24].
- Goal-directed fluid stewardship—monitoring intake–output trends, weight, urine output, and clinical signs of hypovolaemia or overload; communicating deviations. Safety rationale: prevents complications from fluid deficit (hypotension, acute kidney injury) or overload (pulmonary oedema, delayed recovery) [21,25,26].
- Device minimisation—evaluating ongoing need for drains, catheters, and lines each shift; prompting timely removal. Safety rationale: reduces device-related harms (catheter-associated infections, mobility restriction) [7,23,27].
- Reliable discharge teaching—using structured ERAS education tools and teach-back to verify patient understanding of mobilisation, nutrition, analgesia, warning signs, and discharge milestones. Safety rationale: prevents readmissions and emergency visits related to medication errors, activity misunderstandings, or unrecognised complications [13,28,29].

These six safety behaviours form the foundation for the ERAS-aligned nursing practice-development framework presented in this paper. They are nursing-sensitive processes that can be standardised through simulation, observed, assessed, and monitored within ERAS-oriented patient-safety and quality-improvement programmes. Detailed alignment of these behaviours with practice outcomes and assessment strategies is provided in Table 1 (see Domain 1, Section 4.1). In subsequent sections (Domains 1–4), references to “core ERAS safety behaviours” refer to this master definition.

### 3.2. Framework Derivation and Overview

To translate the core ERAS safety behaviours (defined in Section 3.1) into practical educational strategies, we present a four-domain framework that links ERAS clinical pillars to simulation design, assessment, and implementation. The overarching goal is to prepare pre-licensure and early-career medical–surgical nurses to promote early mobility, nutrition, and recovery as safety-critical behaviours; to reduce reliance on opioids via multimodal analgesia; to manage care transitions; and to use patient-centred strategies that support ERAS adherence [30,31].

### 3.3. Domain 1: ERAS Clinical Pillars and Priority Nursing Competencies

Domain 1 establishes the competency foundation by mapping ERAS clinical pillars to observable nursing behaviours that directly influence patient-safety. Table 1 (adapted from ERAS guidelines [7,29,32]) aligns each ERAS element with a safety-focused clinical aim, an ERAS-aligned nursing practice outcome, and example assessment indicators. These indicators, such as time to first ambulation, proportion of non-opioid analgesic doses, documentation of teach-back, and catheter days are nursing-sensitive process measures that can be tracked for quality improvement. Table 1 illustrates how Domain 1 ERAS clinical pillars are translated into ERAS-aligned nursing practice and learning outcomes, together with assessment strategies that make these safety-relevant behaviours observable for pre-licensure and early-career nurses.

**Table 1:** Alignment of ERAS elements with nursing practice outcomes and assessment strategies.

ERAS Element	Safety-Focused Clinical Aim (Nursing Role)	ERAS-Aligned Nursing Practice Outcome	Example Assessment/Monitoring Indicators	References
Early Mobilisation	Promote safe, progressive ambulation to reduce immobility-related complications (venous thromboembolism, atelectasis, deconditioning) and support timely recovery.	Initiates and documents early mobilisation within ERAS timeframes, tailors assistance to patient risk, and reinforces the physiological rationale for ambulation.	Time to first ambulation; number of assisted walks per shift; documentation of mobility level and tolerance; variance reports when mobilisation is delayed.	[5,7,13,18,19,23]
Multimodal Pain Management	Optimise pain control while minimising opioid-related adverse events (ileus, oversedation, delirium, respiratory depression).	Performs structured pain assessments, implements non-opioid and non-pharmacologic strategies first line, and escalates opioids judiciously within the multimodal plan.	Proportion of doses from non-opioid options; pain scores before/after interventions; documentation of adverse effects and actions taken; checklist of multimodal components delivered.	[5,7,20,21,22]
Early Oral Feeding	Support timely return of gut function and reduce infection and ileus through safe, early oral intake.	Assesses readiness to advance diet, initiates ordered early feeding, monitors tolerance (nausea, vomiting, distension), and escalates concerns promptly.	Time to first oral intake; percentage of patients receiving feeds by POD-1; documentation of tolerance and escalation; variance when NPO status persists without indication.	[5,7,23,33]
Preoperative Education	Prepare patients and families for ERAS expectations, thereby reducing anxiety, delays, and non-adherence that compromise safety.	Uses structured ERAS teaching tools and teach-back to explain mobilisation, analgesia, diet advancement, and discharge milestones.	Completion of ERAS education checklist; documentation of teach-back; patient-reported understanding of ERAS plan; linkage to post-op adherence.	[5,7,13,32,34]
Fluid Management	Maintain euvolaemia and prevent complications from fluid overload or deficit (hypotension, acute kidney injury, pulmonary oedema).	Monitors intake–output trends, weight, urine output, and clinical signs; communicates deviations from goal-directed fluid plans to the team.	Accuracy of intake and output charting; timely reporting of deviations; concordance between nursing documentation and ordered fluid strategy.	[5,7,21,26,35]
Avoidance of Drains/Catheters	Reduce device-related infections and support mobility by limiting unnecessary drains and urinary catheters.	Evaluates ongoing need for devices each shift, prompts timely removal, and prepares patient for mobility once devices are discontinued.	Catheter and drain days; documentation of daily necessity assessment; time to removal versus ERAS target.	[5,7,27,36]
Discharge Planning from Day 1	Reduce readmissions and delays by aligning daily care with clearly defined discharge criteria from the first post-op day.	Uses ERAS discharge checklists to set daily recovery goals, updates the team on barriers, and documents progress toward criteria.	Use of standardised discharge criteria; documentation of barriers; readmission or unplanned emergency department visit rates related to discharge issues.	[5,7,13,32]
Interprofessional collaboration	Coordinate care across disciplines so that ERAS milestones (mobility, diet, analgesia) occur on schedule.	Uses structured tools (e.g., SBAR, huddles, rounds) to communicate patient status and advocate for ERAS adherence with surgeons, anaesthesia, physiotherapy team, dietetics, and pharmacy.	Quality of handoff ratings; documentation of interprofessional plans; resolution of variances identified in team huddles.	[13,29,37,38]
Patient-Centred Care	Align recovery goals with what matters to the patient, improving adherence and early identification of concerns.	Co-creates daily recovery goals with patients/families, documents preferences and worries, and integrates these into ERAS plans.	Documentation of patient-stated goals; patient-reported engagement; correlation between goals and delivered care.	[5,13,29,39,40]
Minimising Opioid Use	Reduce opioid exposure and related harms while maintaining functional pain control that may help mobilisation.	Explains risks and benefits of opioids, prioritises non-opioid options, monitors for early signs of toxicity, and adjusts the plan with the team.	Opioid morphine milligram equivalents per patient; documentation of opioid-related adverse effects; alignment of analgesia regimen with multimodal protocol.	[5,7,20,21,22]

Note. ERAS elements and their linked ERAS-aligned nursing practice and learning outcomes and assessment strategies were informed by ERAS guidelines and prior educational and implementation studies.

In addition, Table 2 maps the ERAS-related nursing competencies to the AACN Essentials [41], providing educators and nurse managers with a direct link between regulatory standards and ERAS-specific practice outcomes. This mapping supports curriculum integration and competency validation.

### 3.4. Domain 2: Scenario and Modality Design—A Worked Example

Domain 2 translates the competencies from Domain 1 into concrete scenario and modality choices that make recovery and safety behaviours observable and assessable. The practice-development goals focus on strengthening ERAS-aligned assessment, coordination, and advocacy skills. Participants are expected to evaluate patient readiness for early oral intake and mobilisation, conduct comprehensive pain assessments using opioid-sparing strategies, and communicate with the medical or physiotherapy team using structured SBAR techniques [8,44]. The scenario typically involves a primary nurse, a secondary nurse, an observer or charge nurse, and optionally a patient actor or family member [44,45]. The timing structure includes a brief pre-briefing, 15–20 min of active simulation, and a 20–30 min debriefing session [44,46].

Table 3 presents a representative ERAS-aligned simulation scenario centred on a postoperative day (POD) 1 assessment following laparoscopic colorectal surgery. The scenario operationalises the core safety behaviours defined in Section 3: early mobilisation, opioid-sparing multimodal analgesia, early oral feeding, fluid stewardship, device minimisation, and reliable discharge teaching. Participants are exposed to a typical postoperative deviation, an inadvertent NPO order, which must be clarified using SBAR, thereby rehearsing detection and correction of safety-threatening pathway drift. Importantly, this scenario is a conceptual example developed from ERAS guidelines and the authors’ collective expertise; it has not yet been piloted with learners or formally validated. We present it as an illustrative template that institutions can adapt and evaluate locally.

Table 4 provides a time-stamped overview of key cues and corresponding expected participant actions during the scenario, illustrating how each safety behaviour can be observed and assessed in real time [8,44].

To make the scenario operational rather than merely illustrative, each learning objective was linked conceptually to a corresponding competency, observable behaviour, and assessment approach. In practice, identification and escalation of the NPO-order discrepancy can be assessed through an ERAS checklist item and relevant Lasater Clinical Judgement Rubric domains such as noticing and responding; safe early mobilisation can be evaluated through completion of prerequisite safety checks, patient preparation, and successful assisted ambulation; implementation of the multimodal analgesia plan can be assessed through medication decision-making and pain reassessment; and teach-back-based patient education can be evaluated through confirmation of patient understanding and documentation quality. Any associated benchmarks, such as identifying the NPO discrepancy during the initial phase of the scenario, initiating ambulation before scenario completion, or completing teach-back before debriefing, should be interpreted as illustrative local performance targets rather than validated standards.

### 3.5. Domain 3: Assessment and Feedback Strategies for ERAS Safety and Reliability

Domain 3 specifies assessment and feedback strategies, including tools to assess clinical judgement (Lasater Clinical Judgement Rubric, LCJR), handoff quality, and ERAS adherence using structured checklists. Table 5 summarises core assessment tools that can be used to evaluate ERAS-specific performance, support structured debriefing, and provide feedback data that nurse managers can incorporate into local quality dashboards [50,51].

**Table 5:** Assessment and feedback tools for ERAS-aligned nursing simulation.

Tool	Description/ERAS Safety and Quality Improvement Focus
LCJR	Assess noticing, interpreting, responding, and reflecting on ERAS-related safety cues and recovery milestones (e.g., early mobilisation, opioid stewardship, fluid status, device use) during simulation, providing a structured view of clinical judgement that can inform unit-level competency validation.
Video-Based Debriefing	Replay key segments of the scenario; ask pre-licensure and early-career nurses and staff to identify clinical-reasoning cues, patient-safety risks, and examples of adherence or non-adherence to ERAS protocols, linking observations to desired practice patterns and quality-improvement priorities.
Guided Self-Reflection Questions	Prompt participants to consider which ERAS principles they applied, how they prioritised nursing actions to reduce preventable harm and support reliable recovery, where safety behaviours were missed or delayed, and what they would change in future simulations or clinical practice.

Note. An instructor- and nurse-manager-facing template to support consistent planning, delivery, and documentation of ERAS-aligned simulation sessions as a patient-safety and quality-improvement intervention is provided in Table 6 [7,51,52,53].

**Table 6:** Implementation barriers and phased strategies for ERAS-aligned simulation as a nursing practice-development and patient-safety intervention, corresponding to the short-, medium-, and long-term approaches.

Barrier	Short-Term Practice-Development Strategy (0–6 Months)	Medium-Term Implementation Strategy (6–18 Months)	Long-Term System-Integration Strategy (18+ Months)
Lack of ERAS knowledge among faculty, nurse managers, and clinical educators	Organise focused ERAS patient-safety journal clubs or in-service sessions that highlight recovery pathways, nursing roles, and local safety or outcome data.	Provide CE-accredited courses on ERAS and simulations that emphasise nursing-sensitive safety indicators, care-reliability concepts, and debriefing skills.	Create structured ERAS-simulation training programmes and designate interprofessional ERAS simulation champions who explicitly link simulation activities with unit quality-improvement priorities.
Variation in clinical exposure to ERAS and inconsistency in pathway adherence across sites	Standardise exposure to essential ERAS experiences (e.g., POD-1 colorectal care, early mobilisation, opioid-sparing analgesia) through simulation-based “minimum standard” scenarios.	Align simulations with related courses, unit orientations, and ERAS order sets to reinforce key pathway elements, expected nursing behaviours, and discharge-education practices.	Build formal academic-clinical partnerships to maintain ERAS-aligned simulations, periodically review local adherence and patient-safety data, and co-lead scenario updates when pathways or policies change.
Limited protected time for nurse managers, clinical educators, and faculty to design or update simulations	Use plug-and-play, modular ERAS scenarios and accompanying checklists that can be embedded into existing skills labs, orientations, safety huddles, or in-service sessions.	Develop a shared ERAS simulation repository (cases, cue sheets, debriefing guides) accessible to academic and clinical teams to reduce duplication of effort.	Embed ERAS-simulation planning into standing curriculum and quality-improvement committee agendas so scenarios are routinely reviewed, updated, and scheduled alongside other patient-safety initiatives.
Insufficient simulation equipment or dedicated lab space	Use low-cost modalities (role-playing, paper or virtual cases, bedside drills) to rehearse ERAS safety behaviours such as mobilisation coaching, opioid-sparing pain management, and device-removal timing.	Purchase portable, low-fidelity kits (e.g., mobility aids, incentive spirometers, pain-assessment tools) that support ERAS-focused skills practice on wards or in classrooms.	Seek grants or interdepartmental funding to expand simulation labs and integrate ERAS scenarios into institutional simulation centres used for system-wide patient-safety and high-reliability training.
Limited preparation time for pre-licensure learners and staff participating in ERAS simulations	Provide concise ERAS reading briefs, infographics, or pre-simulation micro-videos that emphasise safety rationales, recovery milestones, and expected nursing actions.	Implement flipped or blended approaches in which core ERAS concepts are completed asynchronously so in-person time focuses on practising high-risk, high-impact behaviours (e.g., escalation of care, teach-back discharge education).	Integrate competency-based evaluation for pre-licensure and early-career nurses with ERAS safety and adherence objectives, linking results to ongoing orientation and annual competency review processes.

Note. Developed as a synthesis of the previously published ERAS and simulation implementation literature to guide nurse managers, ERAS programme leads, and academic partners in planning ERAS-aligned practice-development strategies across short-, medium-, and long-term timeframes [54,55,56].

Structured debriefing including video-assisted review and reflective prompts tied to the core safety behaviours is essential. Debriefing should focus on clinical reasoning in ERAS care, identification of latent safety threats (e.g., conflicting orders), interprofessional communication, and how observed behaviours could be tracked as nursing-sensitive indicators for unit-level quality improvement.

### 3.6. Domain 4: Implementation Tools and Practice-Oriented Integration

**Figure 1:**
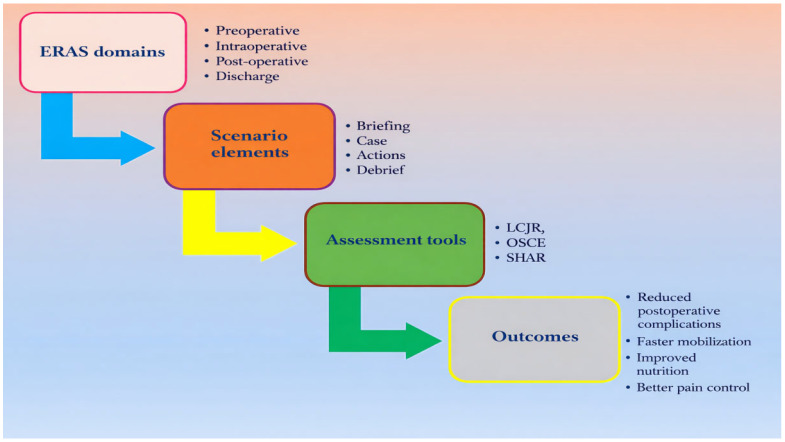
Logic model linking ERAS principles to simulation activities, proximal outputs, and learner/patient outcomes.

**Figure 2:**
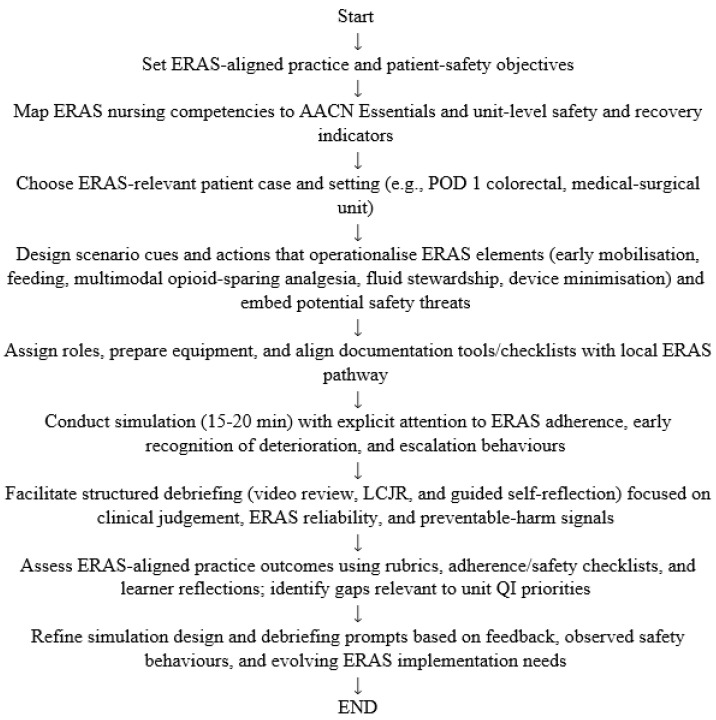
ERAS-aligned nursing practice development and safety-focused simulation workflow for instructors and nurse managers.

**Figure 3:**
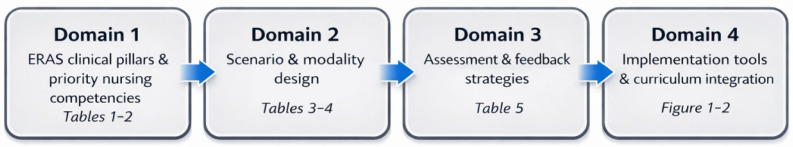
Framework organising ERAS-aligned nursing practice development into four interconnected domains: (1) ERAS clinical pillars and safety-critical nursing competencies (Table 1 and Table 2); (2) scenario and modality design that rehearses ERAS behaviours and exposes latent safety threats (Table 3 and Table 4); (3) assessment and feedback strategies that evaluate clinical judgement, ERAS adherence, and recovery-focused safety behaviours (Table 5); and (4) implementation tools and practice integration that connect simulation outputs to unit-level targets, competency validation, and curriculum planning (Table 6; Figure 2). Arrows indicate the directional flow from Domain 1 to Domain 4, illustrating how ERAS principles are translated through scenario design and assessment into ongoing practice development, monitoring of nursing-sensitive indicators, and long-term sustainability of ERAS-aligned care.

Domain 4 focuses on practical tools that support scalable integration of ERAS-aligned simulation within pre-licensure programmes and unit-based practice-development initiatives [3,57]. Figure 1 (logic model) and Figure 2 (workflow) illustrate how simulation activities link to proximal process outputs (observable safety behaviours) and downstream patient and system outcomes, though these relationships are conceptual and require empirical validation. Figure 3 summarises the four-domain framework visually.

For nursing management, the framework offers a mechanism to align unit-level orientation, competency validation, and quality dashboards with ERAS safety targets. Nurse managers can use Domain 1 indicators (e.g., time to first ambulation, opioid morphine milligram equivalents per patient, documentation of teach-back) as process measures for staff development and performance monitoring. By co-owning scenario priorities and outcome metrics with academic partners, they can use the framework as a practical lever to improve ERAS adherence and reduce variability in postoperative care. The four domains are interdependent: Domain 1 defines what to teach, Domain 2 specifies how to simulate it, Domain 3 provides tools to assess learning, and Domain 4 offers resources to embed the activities within educational and clinical systems. Together, they form a coherent, safety-oriented practice-development model that warrants prospective evaluation.

## 4. Discussion

This paper presents an evidence-informed, four-domain framework for integrating ERAS principles into simulation-based nursing practice development for pre-licensure and early-career nurses. The framework contributes a structured approach to translating ERAS safety behaviours—early mobilisation, opioid-sparing analgesia, early oral feeding, fluid stewardship, device minimisation, and reliable discharge teaching—into observable and assessable simulation activities that are linked conceptually to patient-safety and quality-improvement priorities. Importantly, the framework should be interpreted as a practice-oriented, evidence-informed model rather than as an empirically validated intervention. Throughout this paper, terms such as “framework”, “template”, “indicator”, and “implementation tool” refer to conceptual propositions derived from narrative review and expert-informed refinement. They should not be interpreted as evidence that the framework improves clinical outcomes or as a validated implementation package. References to potential patient-safety benefits are therefore intended as plausible hypotheses and candidate targets for future empirical evaluation, not as demonstrated effects.

### 4.1. Relationship to Existing ERAS and Simulation Frameworks

Existing ERAS publications have primarily focused on pathway components, staff training, or implementation challenges [58,59], whereas established simulation standards focus on simulation design, facilitation, and debriefing across broad clinical content areas [44,52]. The present framework does not replace these bodies of work; rather, it integrates them. Specifically, it uses ERAS guidance to define safety-critical postoperative content, AACN Essentials to anchor competency mapping [41], and established assessment approaches such as the Lasater Clinical Judgement Rubric and structured debriefing to support educational evaluation [44,52].

Compared with prior undergraduate simulation reports, which have rarely treated ERAS as a central organising content area [60], the present framework is more explicitly ERAS-specific. It links competency development with nursing-sensitive recovery behaviours and candidate quality indicators, such as time to first ambulation, opioid exposure, catheter duration, and documentation of teach-back [41,61]. In this sense, the framework extends existing simulation literature by adding content specificity and implementation relevance, while remaining dependent on broader simulation design principles already established in the field [44,52].

The framework is also supported by established learning theories. Experiential learning theory [62,63] aligns with the cycle of concrete simulation experience, reflective debriefing, and active experimentation. Cognitive load theory [64] supports the progressive introduction of clinical complexity, allowing learners to coordinate analgesia, mobilisation, and fluid goals under guided conditions. Benner’s novice-to-expert framework [65,66] further suggests that repeated exposure to ERAS-aligned scenarios may support progression from rule-based task execution toward more anticipatory and safety-focused clinical reasoning. These theoretical foundations strengthen the rationale for simulation as a mechanism for ERAS practice development, even though they do not in themselves validate the proposed framework.

### 4.2. What Is Novel Versus Adapted

The novelty of the present paper is therefore integrative and translational rather than methodological. The framework brings together ERAS content, pre-licensure and early-career nursing preparation, competency mapping, a worked postoperative day-one colorectal scenario, and nursing-sensitive quality indicators within one coherent model. This combination is not commonly presented in prior ERAS or simulation literature, where educational content, implementation strategy, and competency assessment are often discussed separately [58,59]. Novel elements of the framework include explicit mapping of ERAS pillars to the AACN Essentials, an adaptable competency-based POD-1 colorectal scenario template, and linkage of simulation activities to candidate patient-safety and quality indicators.

At the same time, several elements of the framework are deliberately adapted rather than new. The ERAS content priorities are drawn from published ERAS guidance [58,59]; the competency orientation is anchored in AACN Essentials [41]; and the assessment and facilitation approaches build on established simulation literature and standards, including the Lasater Clinical Judgement Rubric, structured debriefing, and INACSL-aligned design principles [44,52]. Accordingly, the contribution of this paper lies less in inventing new educational theory or assessment methods than in organising established elements into an ERAS-specific, nursing-focused, and implementation-oriented structure.

### 4.3. Practical Implementation Constraints and Practice Implications

Although the framework is intended to support practical adoption, implementation will depend on local faculty expertise, simulation capacity, curricular time, interprofessional access, and the maturity of local ERAS programmes. Institutions without established ERAS pathways may need to adapt scenario triggers, order sets, expected nursing actions, and candidate performance benchmarks substantially. Similarly, indicators such as time to first ambulation, opioid exposure, catheter days, or discharge-teaching reliability may not be routinely measured or directly comparable across settings. These contextual differences mean that the framework should be used as a flexible template rather than a practice-ready bundle.

From a practical perspective, the framework offers several potential uses. For educators, it provides practice-oriented competency maps and tools (Table 1 and Table 2), a conceptual scenario template (Table 3 and Table 4), and assessment tools (Table 5) that can be adapted to local curricula. For nurse managers and ERAS programme leads, it offers a way to align simulation activities with unit-level quality indicators, such as early mobilisation rates, opioid-related adverse events, catheter duration, and discharge readmission patterns, thereby “supporting the incorporation of simulation-derived competencies into broader quality-improvement efforts” [63,67]. For frontline nurses, rehearsing core ERAS safety behaviours in a controlled environment may support confidence, clinical judgement, and interprofessional communication, although these outcomes require empirical confirmation [68].

Practical uptake is nevertheless likely to be influenced by several barriers, including variable faculty preparedness, inconsistency in ERAS exposure across clinical sites, time and resource constraints, and limited access to simulation equipment [54,55,56,69]. Short-term responses may include use of plug-and-play scenarios in existing skills laboratories, ward-based drills, or safety huddles. Medium-term strategies may involve shared scenario repositories, faculty development workshops, and closer alignment of scenarios with ERAS order sets and local recovery targets. Longer-term integration may require incorporation of ERAS-aligned simulation into curriculum planning, unit orientation, competency validation, and quality committee agendas. Table 6 summarises these barriers and phased implementation strategies.

A staged approach across pre-licensure preparation and transition to practice may also strengthen feasibility. Early in medical–surgical education, learners may focus on foundational ERAS concepts such as preoperative teaching, mobilisation principles, NPO-status clarification, and basic fluid assessment [69]. Later stages can introduce multimodal analgesia, postoperative day-one recovery management, and discharge readiness. In final-year capstone experiences and new-graduate orientation, learners may engage in more comprehensive interprofessional ERAS case management that includes bedside rounds, SBAR escalation, team coordination, and recovery-focused discharge teaching [11,70,71,72,73]. Complementary strategies such as flipped classroom preparation, pre-simulation, e-modules, and interprofessional participation by pharmacy, physiotherapy, or dietetics learners may further improve realism and scalability [74,75,76,77,78], although these should be viewed as extensions of simulation rather than substitutes for it.

Nurse managers occupy a particularly important position at the interface of education, practice development, and quality improvement in ERAS programmes. The framework may help nursing management map local postoperative quality indicators, such as mobilisation timing, postoperative ileus, opioid consumption, or discharge delays to explicit learner and staff competencies, thereby linking practice-development efforts to service priorities [79]. Many of these indicators are also patient-safety markers, including immobility-related complications [80], opioid-related adverse events [81], device-related harms [82], and failures in discharge education [83]. Example local benchmarks may include timely ambulation, correction of NPO-order discrepancies, teach-back completion, and structured SBAR escalation. However, such benchmarks should be treated as locally adaptable examples rather than validated standards.

### 4.4. Limitations and Implications for Future Empirical Research

The framework has several limitations that must be acknowledged. It is conceptual and has not been empirically tested for effects on patient outcomes such as opioid-related adverse events, immobility-related complications, catheter-associated harms, or readmission rates. The literature review was narrative rather than systematic, and no formal risk-of-bias appraisal was conducted. Expert input strengthened the framework’s clinical and educational relevance, but the absence of a structured consensus process means that it should not be interpreted as consensus-validated guidance. Rather, the framework should be understood as an expert-informed model that requires empirical testing and further refinement in diverse settings. ERAS implementation varies across healthcare systems, surgical populations, and institutional infrastructures, which may affect transferability. In addition, the postoperative day-one colorectal scenario is an illustrative conceptual example that requires local adaptation and evaluation before use as a formal teaching or assessment package. All proposed patient-safety implications should therefore be interpreted as conceptual and hypothesis-generating until confirmed through empirical study.

These limitations point directly to the next phase of work. The immediate need is not further conceptual expansion, but prospective empirical testing. Future studies should examine feasibility, acceptability, and inter-rater reliability of the proposed scenario and assessment approach, together with learner outcomes such as clinical judgement, communication, confidence, and self-efficacy. Longitudinal designs tracking transition-to-practice performance, competency retention, and practice behaviours would be especially valuable. Complementary qualitative work, including reflective journals, focus groups, and debrief transcript analysis, could help clarify how learners and facilitators experience ERAS-aligned simulation and which elements are perceived as most useful.

Only after such educational and implementation testing should patient-level outcomes be examined. Subsequent studies may then explore whether ERAS-aligned simulation is associated with measurable changes in delayed mobilisation, opioid-related adverse events, catheter duration, discharge-education reliability, or preventable readmissions, particularly when linked to unit-level quality dashboards. Until such data are available, the framework should be regarded as a practice-development guide that identifies plausible educational targets, implementation strategies, and candidate evaluation metrics rather than as a proven effectiveness model.

In summary, the proposed framework offers a structured, safety-oriented approach to embedding ERAS principles into pre-licensure nursing preparation and early-career practice development. By linking simulation-derived competencies to candidate quality indicators, it provides a common blueprint for academic-clinical partnerships seeking to strengthen the reliability of postoperative care and reduce preventable harm. However, these potential benefits remain conceptual until tested through rigorous educational, implementation, and outcome research.

## 5. Conclusions

This paper presents an evidence-informed, four-domain framework that embeds ERAS principles within simulation-based nursing practice development for pre-licensure and early-career nurses. The framework organises ERAS safety behaviours—early mobilisation, opioid-sparing analgesia, early oral feeding, fluid stewardship, device minimisation, and reliable discharge teaching—into observable simulation activities, assessment approaches (e.g., the Lasater Clinical Judgement Rubric), and implementation considerations relevant to nurse educators and managers. Unlike prior publications that focus primarily on staff training or general perioperative competencies, the framework is ERAS-specific, maps to AACN Essentials, and proposes candidate links between simulation activities and unit-level quality indicators such as ambulation timing, opioid exposure, and discharge-education reliability.

For faculty and nurse managers, the framework may help align pre-licensure preparation, orientation, and competency validation with patient-safety and quality-improvement priorities. The included POD-1 colorectal scenario, competency maps, and implementation strategies are intended as illustrative, practice-oriented resources rather than validated implementation tools. As such, they may support local adaptation by academic and clinical partners seeking to strengthen ERAS-aligned nursing preparation.

The framework is conceptual and has not been empirically tested for effects on patient outcomes. Its propositions derive from a narrative literature review and iterative expert input rather than a systematic review or formal consensus process. Accordingly, the framework should be interpreted as an expert-informed practice-development guide that warrants prospective evaluation and refinement. Future research should examine its feasibility, acceptability, and educational effects, and only subsequently explore whether ERAS-aligned simulation is associated with patient-level outcomes such as opioid-related adverse events, immobility-related complications, device-related harms, and preventable readmissions. Until such evidence is available, the framework should be viewed as a structured conceptual foundation for advancing ERAS-aligned nursing education and practice development rather than as a validated effectiveness model.

## Figures and Tables

**Table 2:** ERAS-related nursing competencies mapped to AACN Essentials and ERAS-aligned practice outcomes.

ERAS Competency	Mapping to AACN Essentials	ERAS-Aligned Practice and Learning Outcome	References
Early mobilisation and functional recovery	Essential 2: Patient-Centred Care	Initiate and document safe early ambulation and mobility plans for postoperative patients in collaboration with physiotherapy and the wider team, aiming to reduce immobility-related complications and support functional recovery.	[13,29,42]
Pain and symptom management using opioid-sparing strategies	Essential 1: Knowledge for Nursing Practice	Implement and evaluate multimodal, opioid-sparing pain-management plans; monitor analgesic effectiveness and adverse effects; escalate concerns to maintain comfort while minimising opioid-related harm.	[20,21,22]
Patient education and discharge readiness	Essential 8: Informatics and Technology	Use standardised ERAS education tools and electronic prompts, applying teach-back to verify understanding of mobilisation, nutrition, analgesia, and warning signs, and document discharge readiness in the health record.	[28,29]
Interprofessional communication for ERAS reliability	Essential 6: Interprofessional Partnerships	Use structured communication approaches (e.g., SBAR) to coordinate with surgeons, anaesthesia, physiotherapy, pharmacy, and other team members about ERAS goals, report deviations, and advocate for protocol adherence and safe discharge.	[43]

Note. Competencies and mappings were developed based on the AACN Essentials and prior ERAS-related nursing and patient-safety studies.

**Table 3:** Design summary for a POD-1 colorectal ERAS simulation scenario to rehearse nursing safety behaviours and support ERAS pathway reliability.

Component	Details/Example
Simulation Title	Post-Op Day 1: ERAS Early Mobilisation and Opioid-Sparing Pain Management.
Level of Learners	Pre-licensure and early-career nurses assigned to medical–surgical or colorectal ERAS units.
Estimated Duration	10 min prebrief, 20 min scenario, 30 min debrief.
Location/Setting	Medical–surgical unit or Post-Anaesthesia Care Unit caring for colorectal patients on an ERAS pathway.
Learning Objectives	Identify the conflict between the active NPO order and the ERAS oral-intake plan during the initial phase of the scenario and escalate it using SBAR communication. Complete a focused postoperative safety assessment and initiate assisted mobilisation using appropriate orthostatic, fall-risk, and analgesia checks. Implement the multimodal pain plan, select and explain non-opioid first-line choices, and reassess pain after intervention. Deliver brief teach-back-based education on early ambulation, oral intake, breathing exercises, and expected recovery milestones, and document the patient’s response.
Patient Profile	Mr. Rao, 58-year-old male, POD-1 after laparoscopic sigmoid colectomy on a colorectal ERAS pathway.
Clinical Status	Stable vital signs; pain 5/10; hesitant to ambulate; chart shows NPO status mistakenly continued despite ERAS orders for early oral fluids, creating risk for delayed recovery and pathway drift.
Roles	Primary nurse (lead), secondary nurse/observer, family member or support person (optional), physiotherapy consult (played by facilitator or learner), with charge nurse or team leader optionally observing system issues.
Key Equipment	Vital signs monitor, medication administration record, walker, incentive spirometer, mock chart/electronic health record view, dietary tray card or fluids order sheet.
Initial Cues	Patient in bed, anxious about walking and asking when eating can resume. Orders include early ambulation and clear fluids as tolerated. Nurse notes that NPO status is still active in the chart, conflicting with ERAS orders.
Expected Student Actions	Use SBAR to clarify and correct the erroneous NPO order with the medical team. Encourage, prepare, and assist the patient with early ambulation while monitoring safety. Administer scheduled non-opioid analgesics and evaluate effectiveness as part of the multimodal pain plan. Educate the patient about ERAS goals (early walking, oral intake, breathing exercises) and document key safety behaviours (time to first ambulation, tolerance of fluids).
Critical Safety Actions	Complete focused pain and safety assessment prior to mobilisation and feeding. Identify and resolve the NPO–ERAS order discrepancy to prevent unnecessary fasting. Implement an opioid-sparing multimodal pain regimen that supports mobilisation. Use SBAR to communicate with physiotherapy and the medical team regarding progress, concerns, and next-step plans.
Debriefing Focus	Clinical reasoning in ERAS care (balancing pain control, mobilisation, and fluid/nutrition goals). Identification and management of latent safety threats (e.g., conflicting orders, pathway drift). Interprofessional communication and escalation for ERAS reliability. How behaviours in the scenario (time to ambulation, correction of NPO order, patient coaching) could be tracked as nursing-sensitive indicators for unit-level quality improvement.
Assessment Tools	Lasater Clinical Judgement Rubric (LCJR), ERAS adherence and safety-behaviour checklist (e.g., ambulation achieved, NPO error corrected, multimodal analgesia implemented), and guided self-reflection worksheet to support feedback for practice development and quality improvement discussions.

Note. Scenario design was informed by ERAS colorectal guidelines and prior simulation reports [7,29,47,48,49].

**Table 4:** Time-stamped cues and expected nursing safety actions.

Time	Cue/Trigger	Expected Nursing Safety Action
0:00	Vital Signs stable but patient lying flat, NPO status still active, reports mild pain	Perform focused surgical-site and safety assessment; review chart/electronic health record for order discrepancies; identify the NPO order that conflicts with ERAS fluids/feeding plans; use SBAR to clarify orders and advocate for timely oral fluids to prevent delayed recovery and related complications.
5:00	Patient hesitant to ambulate, fears pain and possible complications	Provide recovery-focused coaching on benefits of early mobilisation for preventing venous thromboembolism, pulmonary complications, and deconditioning; administer prescribed non-opioid analgesia and appropriate non-pharmacologic comfort measures; reassess pain and readiness to mobilise.
10:00	Orders confirmed for early mobilisation and oral intake	Assist ambulation with walker using fall-prevention and orthostatic-safety checks; initiate or advance oral intake per clarified ERAS orders; monitor and document vital signs, pain, and tolerance to activity/fluids as nursing-sensitive indicators of ERAS adherence.
15:00	Call from physiotherapy team for mobility handoff and collaborative planning	Use SBAR to update physiotherapy team on mobility status, pain control, oral-intake tolerance, and any safety concerns; agree on shared mobility goals and next-step plan; ensure documentation of the interprofessional plan and continued mobilisation strategy.

Note. Time points represent key decision moments used to observe ERAS-aligned nursing safety behaviours, order reconciliation, early mobilisation, multimodal analgesia, and interprofessional SBAR handoff, that can conceptually be tracked as process indicators for unit-level patient-safety and quality-improvement efforts.

## Data Availability

No new data were created or analysed in this study.

## Abbreviations

AACN	American Association of Colleges of Nursing
CINAHL	Cumulative Index to Nursing and Allied Health Literature
ERIC	Education Resources Information Centre
ERAS	Enhanced Recovery After Surgery
INACSL	International Nursing Association for Clinical Simulation and Learning
LCJR	Lasater Clinical Judgement Rubric
MEDLINE	Medical Literature Analysis and Retrieval System Online
POD	Postoperative Day
SBAR	Situation, Background, Assessment, Recommendation
NPO	“Nil Per Os” meaning “nothing by mouth”
